# Association of intrauterine platelet-rich plasma infusion before embryo transfer with endometrial response and live birth: a cohort study

**DOI:** 10.3389/fendo.2026.1863693

**Published:** 2026-08-26

**Authors:** Xiao Yuhong, Wang Bolun, Wang Xiliang, Yan Li, Yu Tingting, Xiu Yinling, Yuexin Yu, Ma Xiangwei

**Affiliations:** Department of Reproductive Medicine, General Hospital of Northern Theater Command, Shenyang, China

**Keywords:** assisted reproductive technology, embryo transfer, endometrial thickness, *in vitro* fertilization, live birth, platelet-rich plasma, recurrent implantation failure, thin endometrium

## Abstract

**Background:**

Intrauterine platelet-rich plasma (PRP) has been proposed as an adjunctive intervention for patients with thin endometrium or recurrent implantation failure before embryo transfer, but evidence remains limited by heterogeneous protocols and small studies.

**Objective:**

To evaluate whether intrauterine PRP infusion before embryo transfer is associated with improved endometrial response and pregnancy outcomes, particularly live birth per embryo-transfer (ET) cycle.

**Methods:**

This retrospective cohort included 620 ET cycles, comprising 310 cycles exposed to intrauterine PRP before ET and 310 matched control cycles without PRP exposure. The primary outcome was live birth per ET cycle. Secondary outcomes included biochemical pregnancy, clinical pregnancy, ongoing pregnancy at 12 weeks, ectopic pregnancy, multiple pregnancy, endometrial-thickness response, triple-line endometrial pattern, conditional pregnancy progression, and delivery/neonatal outcomes. Pregnancy outcomes were analyzed using unadjusted and adjusted comparisons and multivariable models.

**Results:**

The PRP and control groups were similar in age, BMI, baseline endometrial thickness, endocrine parameters, frozen ET status, ICSI use, and single-embryo transfer. PRP-treated cycles had more prior IVF/ICSI cycles, clinician-recorded recurrent implantation failure, history of cavity-distorting fibroid, and history of hydrosalpinx. Baseline and pre-procedure endometrial thickness were similar between groups, but post-procedure endometrial thickness, ET-day endometrial thickness, and triple-line pattern were more favorable in the PRP group. In unadjusted analyses, PRP was associated with higher clinical pregnancy, ongoing pregnancy at 12 weeks, and live birth. Live birth occurred in 82/310 PRP cycles (26.5%) and 53/310 control cycles (17.1%) (risk ratio [RR], 1.55; 95% CI, 1.14–2.10; P = 0.005). After multivariable adjustment, PRP remained associated with live birth (adjusted RR, 1.55; 95% CI, 1.12–2.15; P = 0.009). Sensitivity analyses were directionally consistent.

**Conclusion:**

In this cohort study, intrauterine PRP infusion before ET was associated with improved endometrial development and higher rates of clinical pregnancy, ongoing pregnancy, and live birth.

## Introduction

Assisted reproductive technology (ART) has substantially improved the treatment of infertility, yet embryo implantation remains a major limiting step in achieving live birth (1, 2). Successful implantation requires not only a competent embryo but also a receptive endometrium, adequate vascularization, and coordinated embryo–endometrial signaling (2). This is especially relevant in patients with recurrent implantation failure (RIF), a clinically heterogeneous condition for which the European Society of Human Reproduction and Embryology recently emphasized individualized assessment based on the expected cumulative probability of implantation (3). Because live birth remains the most meaningful endpoint for patients and clinicians, studies of implantation-targeted interventions should ideally evaluate live birth per embryo-transfer cycle using standardized ART terminology (1, 4).

Among endometrial factors, inadequate endometrial development is one of the most assessable and potentially modifiable barriers to implantation (2, 5–7). Although endometrial thickness is an imperfect surrogate for receptivity, multiple large cohort and meta-analytic studies have shown that thinner endometrium is associated with lower implantation, clinical pregnancy, ongoing pregnancy, and live-birth rates after IVF and embryo transfer (5–7). In a large analysis of more than 40,000 embryo transfers, clinical pregnancy and live-birth rates declined as endometrial thickness decreased below approximately 8 mm in fresh IVF-ET cycles and below approximately 7 mm in frozen-thawed embryo-transfer cycles (6). These findings have made persistently thin or suboptimally developing endometrium a frequent clinical reason for cycle postponement, cancellation, or use of adjunctive therapies (5–8).

Platelet-rich plasma (PRP) has emerged as a biologically plausible intrauterine adjunct for patients with thin endometrium, RIF, or both (9–15). PRP is an autologous blood-derived product enriched in platelets and platelet-associated mediators that may influence tissue repair, angiogenesis, stromal remodeling, cellular proliferation, and local inflammatory signaling (11, 16, 17). Translational work supports this rationale; transcriptomic profiling of human endometrial stromal cells treated with autologous PRP has suggested changes in pathways related to cell growth, tissue regeneration, inflammatory response, and antimicrobial effects (16, 17). These translational findings support the biological plausibility of PRP as a candidate adjunct in cycles with suboptimal endometrial development, although they do not in themselves establish clinical efficacy (11, 16, 17).

Clinical evidence for intrauterine PRP is encouraging but not yet definitive. Recent systematic reviews and meta-analyses have reported that intrauterine PRP may improve endometrial thickness and reproductive outcomes in women undergoing ART, particularly those with thin endometrium or previous implantation failure (9, 12–14, 18). A 2024 meta-analysis of randomized controlled trials in women with thin endometrium also suggested that intrauterine autologous PRP may improve endometrial thickness and fertility outcomes (12). However, a recent critical appraisal cautioned that the existing literature still does not justify routine PRP use for all patients with RIF, underscoring the need for better-characterized cohorts and clinically meaningful endpoints (8, 19).

Important gaps therefore remain. Many prior studies were small, focused primarily on endometrial thickness or early pregnancy outcomes, and provided limited information on live birth (4, 9, 12–14, 19). In addition, because PRP is commonly offered to patients with poorer baseline prognosis, unadjusted comparisons may be affected by confounding by indication (8, 19). There is a need for larger real-world studies that combine endometrial-response data with pregnancy outcomes, use live birth as a central clinical endpoint, and evaluate whether observed associations persist after adjustment for baseline reproductive and cycle-level prognostic factors (4, 8, 19).

The present cohort study was designed to address these gaps. We evaluated embryo-transfer cycles exposed or not exposed to intrauterine PRP before embryo transfer, with the primary clinical outcome of live birth per embryo-transfer cycle. Secondary outcomes included biochemical pregnancy, clinical pregnancy, ongoing pregnancy, ectopic pregnancy, multiple pregnancy, conditional pregnancy progression, and delivery/neonatal outcomes. We also assessed endometrial-thickness trajectory and endometrial pattern as intermediate response measures. Accordingly, in this retrospective cohort we examined whether intrauterine PRP exposure before embryo transfer was associated with more favorable endometrial development and a higher probability of live birth, particularly in cycles with suboptimal pre-transfer endometrial thickness.

## Methods

### Study design and reporting

This cohort study evaluated pregnancy outcomes following intrauterine platelet-rich plasma (PRP) infusion before embryo transfer (ET). The study compared ET cycles exposed to intrauterine PRP with control ET cycles not exposed to PRP. The report is prepared in accordance with the Strengthening the Reporting of Observational Studies in Epidemiology (STROBE) recommendations for cohort studies. This was a retrospective cohort study conducted on patients who referred to General Hospital of Northern Theater Command, Shenyang, China between February 2020 and June 2025.

### Setting and data source

Data were obtained from the electronic and/or paper-based records of patients undergoing ET at Department of Reproductive Medicine, General Hospital of Northern Theater Command. The dataset included demographic characteristics, reproductive history, infertility-related history, uterine and endometrial findings, laboratory parameters, embryo-transfer characteristics, PRP exposure details, endometrial response variables, pregnancy outcomes, and delivery/neonatal outcomes when available. Patients with missing data were followed up by phone and only the patients with complete information were kept for analysis. The unit of analysis was the embryo-transfer cycle (4). Therefore, outcomes were calculated per ET cycle. Because some women contributed more than one ET cycle to the analytical cohort, patient identifiers were used to quantify within-patient clustering.

### Participants

Eligible cycles were ET cycles in which sufficient baseline, intervention/exposure, endometrial, and pregnancy-outcome data were available. Cycles were classified into the PRP group if intrauterine PRP infusion was performed before ET and into the control group if ET was performed without intrauterine PRP.

Cycles were eligible if they met all of the following criteria: 1- Women undergoing fresh or frozen ET during the study period; 2- Available documentation of PRP exposure status; 3- Available pregnancy-outcome follow-up from biochemical pregnancy testing to live birth; 4- Available key baseline and cycle-level variables, including age, BMI, endometrial thickness, ET type, and number of embryos transferred.

Cycles were excluded if they had: 1- Missing PRP exposure status; 2- Cancelled ET after PRP for personal or administrative non-medical reasons, including patient withdrawal from proceeding with the planned embryo-transfer cycle after PRP administration.; 3- Missing primary outcome data; 4- Major uterine anomaly incompatible with embryo implantation, if recorded; 5- Use of non-standard adjunctive intrauterine intervention at the same cycle, if recorded; 6- Missing related embryo-transfer information.

### Exposure and comparator

The exposure of interest was intrauterine infusion of autologous PRP before ET. In the PRP group, procedural variables included PRP type, indication, infusion volume, number of PRP infusions, interval from the last PRP infusion to ET, platelet concentration relative to baseline, and adverse events. PRP type was categorized as leukocyte-rich PRP or pure PRP according to the recorded preparation method. Primary indication for PRP was categorized as thin endometrium, recurrent implantation failure (RIF), combined thin endometrium and RIF, or unknown. In 56/310 PRP-treated cycles (18.1%), indication could not be determined because the source record did not contain a structured or sufficiently specific indication entry.

Autologous venous blood (10–20 mL) was collected from each patient under aseptic conditions in tubes containing sodium citrate according to the center’s standard clinical protocol. Briefly, PRP was prepared by double-spin centrifugation at 200 ×g for 12 minutes, followed by second centrifugation at 1600 ×g for 8 minutes. The final PRP product was activated using calcium chloride before injection. A volume of approximately 0.5–1.0 mL was infused into the uterine cavity using a sterile intrauterine catheter under transabdominal ultrasound guidance. PRP infusion was performed 1–2 times before ET, according to endometrial response and clinician decision.

For quality characterization, the available PRP variables included relative platelet concentration vs baseline and leukocyte content. Leukocyte-rich PRP and pure PRP were classified according to the center’s laboratory processing definition based on leukocyte content. Sterile processing was performed according to the center’s standard clinical/laboratory protocol, including sterile blood draw, closed system handling, sterile catheterization and visual inspection. Prophylactic antibiotics were routinely used. Patients were monitored for immediate and short-term adverse events including pain, bleeding, cramping, infection symptoms and vasovagal symptoms, and were recorded in the clinical record.

The control group was selected from embryo-transfer cycles performed during the same study period in which no intrauterine PRP infusion was administered before embryo transfer. Eligible control cycles were required to meet the same inclusion and exclusion criteria as PRP-exposed cycles, including availability of baseline demographic variables, endometrial measurements, embryo-transfer characteristics, and pregnancy outcome follow-up through live birth. Because all eligible PRP-exposed cycles were included, control cycles were selected from the pool of eligible non-PRP cycles using frequency matching to achieve a 1:1 ratio according to age category, baseline endometrial-thickness category, and number of prior embryo-transfer cycles. Matching was performed at the group level rather than as individual pair matching, according to prespecified strata of maternal age (<28 & ≥28 years old), baseline endometrial thickness (< & ≥ 7mm), and number of prior embryo-transfer cycles (≤1 cycle & >1 prior cycle). Cycles without known history of ET were included in the ≤1 prior ET-cycle stratum. Within each matching stratum, when more than one eligible control cycle were available within a matching stratum, controls were randomly selected according to computer−generated random number. Outcome variables were not used as matching variables and were not reviewed during the control-selection step. The selection was performed by MX using the prespecified matching framework before the final outcome analysis dataset was assembled. Control cycles were managed according to the center’s standard embryo-transfer protocol during the same calendar period.

### ART procedures and embryo transfer

ART procedures included IVF, ICSI, fresh ET, and frozen ET, as clinically indicated. All cycles involved autologous oocytes. According to the International Glossary on Infertility and Fertility Care, ART includes interventions involving *in vitro* handling of oocytes, sperm, or embryos for reproductive purposes, including IVF, ET, ICSI, embryo biopsy, PGT, and cryopreservation.

Embryo transfer was defined as the placement of one or more embryos into the uterus at any embryonic stage after IVF or ICSI, consistent with ICMART terminology. ET characteristics included fresh versus frozen ET, fertilization method, number of embryos transferred, embryo stage at transfer, elective single-embryo transfer, PGT type, euploid embryo transfer status, and whether at least one good morphological-quality embryo was transferred. Embryo morphological quality was assessed by experienced embryologists according to the center’s routine morphology-based grading criteria at the relevant developmental stage before transfer. For the present study, a binary variable was created indicating whether at least one transferred embryo was recorded as morphologically good quality in the embryology record.

Endometrial preparation for frozen ET was performed using natural cycle and modified natural cycle protocols, as indicated. Progesterone was started when endometrial thickness reached ≥7 mm or according to physician discretion. Embryo transfer was performed on day 3 to day 6 according to embryo stage and progesterone exposure duration. Luteal-phase support consisted of intramuscular progesterone or oral dydrogesterone and was continued until pregnancy test or the ultrasound confirmation.

### Variables

Baseline variables included age, BMI, gravidity, parity, prior live births, infertility duration, prior IVF/ICSI cycles, prior ET cycles, prior failed implantations, prior miscarriages, prior uterine surgery, AMH, day-3 FSH, TSH, prolactin, baseline endometrial thickness, pre-procedure endometrial thickness, uterine artery pulsatility index, subendometrial flow vascularization-flow index, PRP indication (including thin endometrium and/or RIF), history of adenomyosis, cavity-distorting fibroid, hydrosalpinx, chronic endometritis, frozen ET status, ICSI fertilization, elective single-embryo transfer, and transfer of at least one good morphological-quality embryo. Thin endometrium was defined as endometrial thickness <7 mm before progesterone initiation or before ET, according to the recorded clinical diagnosis. RIF, based on the institutional definition, was defined as failure to achieve clinical pregnancy after at least three transfers of good morphological-quality embryos in life-time. Recurrent implantation failure (RIF) status was extracted from the clinical record as documented by the treating reproductive specialist. Because complete lifetime embryo-transfer history and detailed embryo-quality information from all prior cycles were not consistently available, we did not independently reconstruct RIF using this uniform definition. History of adenomyosis, cavity-distorting fibroid, hydrosalpinx and chronic endometritis were extracted from the clinical record, including prior imaging, operative history, and clinician diagnosis.

### Outcome definitions

The primary outcome was live birth per ET cycle. Live birth was defined according to ICMART terminology as complete expulsion or extraction of a product of fertilization after 22 completed weeks of gestation, with evidence of life after separation, such as breathing, heartbeat, umbilical cord pulsation, or definite movement. Live-birth rates were reported considering ET cycles as denominator.

Secondary pregnancy outcomes included biochemical pregnancy, clinical pregnancy, ongoing pregnancy at 12 weeks, ectopic pregnancy, and multiple pregnancy. Biochemical pregnancy was defined as pregnancy diagnosed only by detection of beta-hCG in serum or urine. Clinical pregnancy was defined as pregnancy confirmed by ultrasonographic visualization of one or more gestational sacs or other definitive evidence of an intrauterine pregnancy. Ectopic pregnancy was defined and analyzed separately as pregnancy located outside the uterine cavity, diagnosed by ultrasound, surgical visualization, or histopathology. Ongoing pregnancy was defined as a viable pregnancy continuing to 12 weeks of gestation. Multiple pregnancy was defined as the presence of more than one gestational sac or fetus, according to the recorded clinical outcome. Serum β-hCG testing was typically performed 12–14 days after embryo transfer according to the center’s routine follow-up protocol. Clinical pregnancy was typically confirmed by transvaginal ultrasonography 2–4 weeks after positive β-hCG or at 5–7 weeks of gestation, when a gestational sac could be documented. In addition to reporting ectopic pregnancy and multiple pregnancy per ET cycle, we also calculated clinically contextualized rates using secondary denominators. Ectopic pregnancy was additionally reported per biochemical pregnancy, and multiple pregnancy was additionally reported per clinical pregnancy and ongoing pregnancy at 12 weeks.

Endometrial monitoring was performed according to the routine clinical protocol of the center. Irrespective of PRP exposure, patients undergoing embryo-transfer cycle preparation were invited for serial transvaginal ultrasound assessments at predefined routine monitoring stages during the cycle. These assessments generally included baseline evaluation at the beginning of cycle preparation, an interim assessment during endometrial preparation, a subsequent assessment before progesterone initiation, assessment at progesterone start when applicable, and assessment on or near the embryo-transfer day.

In PRP-treated cycles, the pre-procedure endometrial thickness was defined as the ultrasound measurement obtained immediately before the first intrauterine PRP infusion or before the clinical decision to administer PRP. The post-procedure endometrial thickness was defined as the first routine ultrasound measurement obtained after the final PRP infusion and before progesterone initiation. In control cycles, the corresponding pre- and post-procedure measurements were selected from the analogous routine ultrasound monitoring stages within the same cycle-preparation pathway. In routine practice, these monitoring visits were generally scheduled within a narrow clinical window, although exact visit intervals could vary slightly according to patient availability and clinician judgment. Endometrial-response analyses were therefore interpreted as comparisons across corresponding routine monitoring stages.

Pregnancy-progression outcomes were calculated from the original collected data and included progression from biochemical pregnancy to clinical pregnancy, clinical pregnancy to ongoing pregnancy at 12 weeks, ongoing pregnancy to live birth, and biochemical pregnancy to live birth. Delivery and neonatal outcomes among cycles resulting in live birth included gestational age at delivery, birthweight, and number of liveborn infants. Where more than one infant was born, the available recorded birthweight variable reflected the mean birth weight per pregnancy. Other obstetric/neonatal morbidity variables were not uniformly available.

### Sample size

Because this was a retrospective observational cohort using available clinical data, all eligible PRP cycles during the study period were included, and an equal number of eligible non-PRP control cycles were selected according to the prespecified frequency-matching framework. The precision of the estimated associations was assessed using 95% confidence intervals around the primary and secondary effect estimates.

### Statistical analysis

Analyses were performed at the ET-cycle level. Continuous variables were summarized as mean ± standard deviation (SD) or median with interquartile range (IQR), depending on distributional characteristics. Categorical variables were summarized as frequency and percentage. Baseline and cycle characteristics were compared between PRP and control groups. Welch t test was used for approximately normally distributed continuous variables, Mann–Whitney U test for skewed or ordinal variables, Pearson chi-square test for categorical variables, and Fisher exact test when expected cell counts were small. Standardized mean differences (SMDs) were calculated to quantify between-group imbalance, with SMD values >0.10 interpreted as potentially meaningful imbalance.

Endometrial response was evaluated using between-group comparisons at each routine monitoring stage. Mean between-group differences with 95% confidence intervals (CIs) were calculated for continuous endometrial-thickness measures. Change in endometrial thickness from pre-procedure/corresponding interim monitoring stage to post-procedure/corresponding subsequent monitoring stage was compared between groups. An analysis of covariance (ANCOVA) model was used to evaluate the association between PRP and post-procedure endometrial thickness while adjusting for pre-procedure endometrial thickness. A repeated-measures mixed ANOVA was used to evaluate endometrial-thickness changes across five timepoints: baseline, pre-procedure, post-procedure, progesterone start, and ET day. The model included group, monitoring stage, and group × monitoring stage interaction. The group × time interaction was used to test whether the endometrial-thickness trajectory differed between PRP and control cycles. Greenhouse–Geisser correction was applied when appropriate.

Pregnancy outcomes were first compared using unadjusted analyses. Absolute risk differences and risk ratios (RRs) with 95% CIs were calculated for binary outcomes. Pearson chi-square test or Fisher exact test was used for unadjusted group comparisons.

The primary adjusted analysis estimated the association between PRP and pregnancy outcomes using modified Poisson regression with robust standard errors, reporting adjusted RRs and 95% CIs. The primary adjusted model included age, BMI, prior IVF/ICSI cycles, prior ET cycles, thin endometrium, RIF, history of cavity-distorting fibroid, hydrosalpinx, pre-procedure endometrial thickness, number of embryos transferred, fresh/frozen ET status, and PGT type. These covariates were selected based on clinical relevance and observed baseline imbalance. Logistic regression was performed as a secondary adjusted model, reporting adjusted odds ratios (ORs) with 95% CIs. Logistic models used the same covariate set as the primary modified Poisson models.

Subgroup analyses for live birth were performed according to thin endometrium status, RIF status, pre-procedure endometrial thickness <7 mm versus ≥7 mm, single versus multiple embryo transfer, fresh versus frozen ET, and age <35 versus ≥35 years. Within-subgroup RRs were calculated, and formal interaction tests were performed using modified Poisson regression models. Embryo-transfer subgroup analyses were performed separately for fresh and frozen ET cycles. In addition to the primary adjusted model, subgroup-specific adjusted models were estimated for frozen and fresh ET cycles.

Sensitivity analyses were performed to assess the robustness of the primary live-birth finding. These included: unadjusted analysis; primary adjusted modified Poisson regression; logistic regression using the same covariates; exclusion of PRP cycles with unknown indication; exclusion of cycles with hydrosalpinx; exclusion of cycles with history of cavity-distorting fibroid; analyses restricted to frozen ET cycles; analyses restricted to fresh ET cycles; analyses restricted to single-embryo transfer; analyses restricted to cycles with ≥2 embryos transferred; analyses stratified by pre-procedure endometrial thickness <7 mm versus ≥7 mm; analysis restricted to PGT-A cycles; and stabilized inverse probability of treatment weighting (IPTW) with weights truncated at the 1st and 99th percentiles. The propensity-score model included prespecified baseline and cycle-level covariates that could influence both treatment assignment and live birth, specifically: age, BMI, prior IVF/ICSI cycles, prior ET cycles, thin endometrium, clinician-recorded RIF status, history of cavity-distorting fibroid, history of hydrosalpinx, pre-procedure endometrial thickness, number of embryos transferred, embryo-transfer type (fresh/frozen), and PGT type. Stabilized inverse probability of treatment weights were calculated, and extreme weights were truncated at the 1st and 99th percentiles. Covariate balance before and after weighting was assessed using standardized mean differences (SMDs). Overlap and weight distribution were evaluated descriptively and graphically. The weighted association between PRP and live birth was then estimated using a weighted modified Poisson regression model with robust standard errors.

Because some women contributed more than one cycle, patient identifiers were used to quantify within-patient clustering. We report the number of unique women included in the analytical cohort, the distribution of cycles contributed per woman, and whether any women contributed both PRP-exposed and non-PRP cycles. The primary analysis was performed at the cycle level, and a patient-clustered sensitivity analysis was additionally conducted using modified Poisson regression with patient-level cluster-robust standard errors and GEE to account for non-independence of cycles contributed by the same woman. As an additional sensitivity analysis, we repeated the main model after restricting the dataset to women contributing only one cycle.

Among PRP-treated cycles, procedural factors were compared between cycles with and without live birth. These analyses assessed PRP type, PRP volume per infusion, number of infusions, interval from last PRP to ET, platelet concentration, adverse events, and PRP indication. Conditional pregnancy-progression analyses were performed among cycles reaching each prior pregnancy milestone. Delivery and neonatal outcomes were analyzed descriptively among cycles resulting in live birth.

All tests were two-sided unless otherwise specified. A P value <0.05 was considered statistically significant. Since subgroup and sensitivity analyses were exploratory, no formal adjustment for multiple comparisons was applied. Statistical analyses were performed using SPSS version 27 and R version 4.3.3. Modified Poisson regression, robust standard errors, and IPTW analyses were performed in R.

### Data completeness and missing data

**Figure 1:**
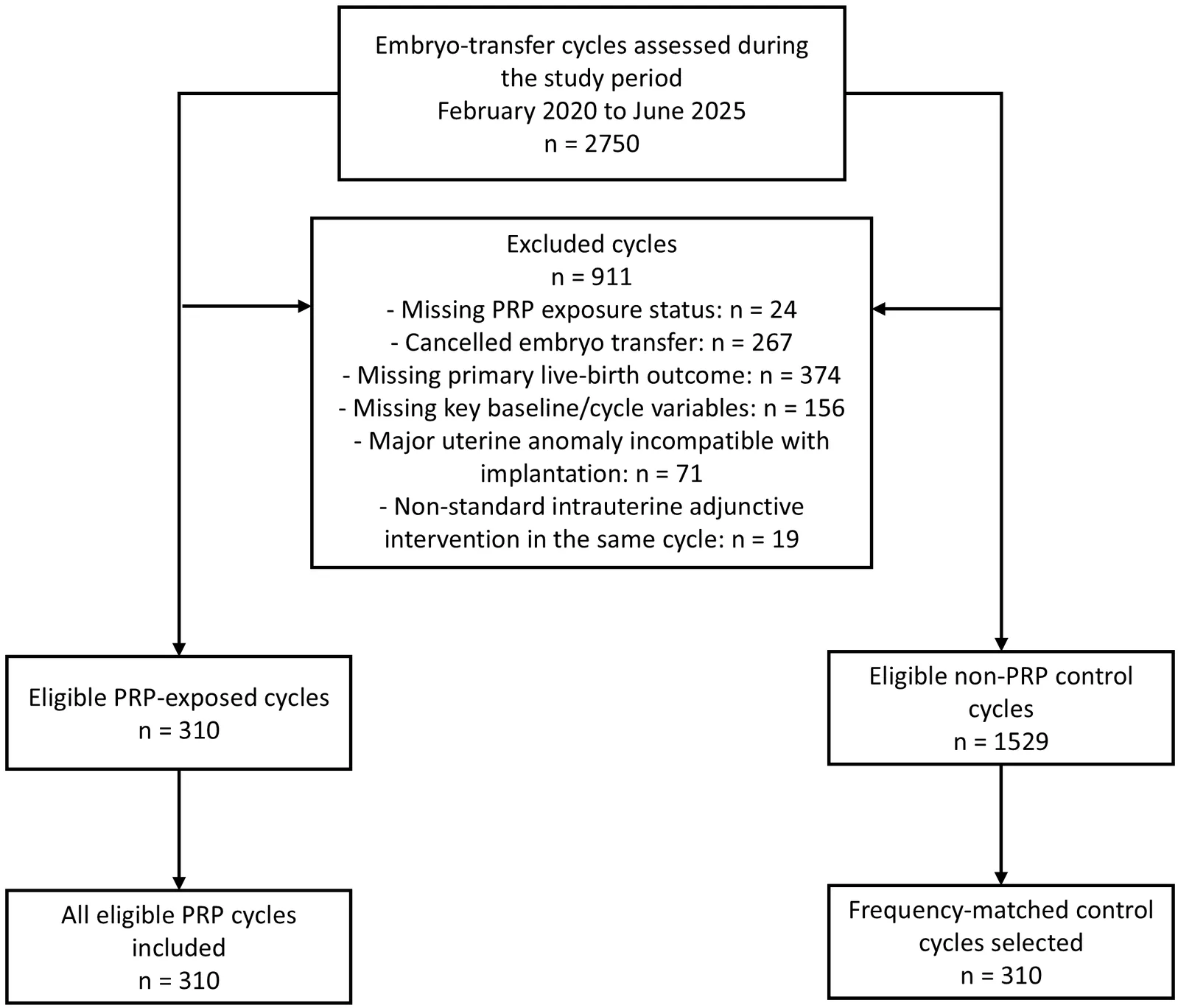
Study flow diagram.

Data completeness was addressed in two stages. First, during cohort assembly from the source database, cycles lacking essential exposure, outcome, or key baseline/cycle information required for the planned analyses were excluded according to prespecified eligibility criteria, as shown in **Figure 1**. These exclusions reflected both missing essential data and other prespecified exclusion criteria.

Second, for cycles that were otherwise eligible but had incomplete follow-up for pregnancy outcome ascertainment, structured telephone follow-up was performed using the contact information recorded in the medical record. Telephone follow-up was used only to ascertain follow-up outcome status including pregnancy and live-birth outcomes. Because part of the follow-up information was obtained by telephone, the possibility of recall or reporting error is acknowledged as a limitation.

After application of the eligibility criteria and completion of outcome ascertainment where possible, the final analytical cohort had no material residual missingness in PRP exposure, the primary live-birth outcome, or the covariates used in the multivariable models. Therefore, the primary analysis used a complete-case approach within the final analytical cohort. Because excluded cycles frequently lacked essential exposure, outcome, or covariate data by definition, they were not suitable for an included-versus-excluded comparison across all major prognostic factors.

### Bias and confounding

No randomization or quasi-randomization was performed at any stage; control selection was conducted retrospectively during cohort assembly using prespecified group-level frequency-matching strata. Because PRP was not randomly assigned, confounding by indication was considered the main potential source of bias. Patients receiving PRP were expected to have a higher baseline burden of implantation-related risk factors, including thin endometrium and/or RIF. To address measured confounding, baseline characteristics were compared between groups using P values and SMDs, multivariable models were adjusted for clinically relevant covariates and imbalanced baseline variables, and IPTW was performed as a sensitivity analysis. However, residual confounding from unmeasured variables could not be excluded.

### Ethical considerations

The study protocol was reviewed and approved by the Ethics Committee of General Hospital of Northern Theater Command on April 2025 under approval number [Y (2025) 077]. Because the study used retrospective de-identified clinical data, the requirement for written informed consent was waived.

## Results

### Study population and baseline characteristics

Of the 2,750 embryo-transfer cycles screened during the study period, exclusions occurred during prespecified cohort assembly for missing essential exposure/outcome/covariate information and other protocol-defined reasons (**Figure 1**). The analytical cohort was then restricted to cycles with complete information required for the planned analyses after outcome follow-up completion where possible. Eligible embryo-transfer cycles with complete baseline, exposure, endometrial-response, embryo-transfer, and live-birth outcome data were identified. All 310 eligible cycles exposed to intrauterine PRP before embryo transfer were included. Of 1,529 eligible non-PRP cycles, 310 were selected as frequency-matched controls according to prespecified strata of age, baseline endometrial thickness, and prior ET cycles (**Supplementary Table 5**).

A total of 620 embryo-transfer cycles were included in the analysis, comprising 310 cycles in the control group and 310 cycles in the intrauterine platelet-rich plasma (PRP) group. These 620 ET cycles contributed by 582 unique women. Most women contributed one cycle (544/582, 93.5%), while 38/582 (6.5%) contributed two cycles; no woman contributed more than two included cycles. Six women contributed both PRP and control cycles. At the cycle level, 44/310 (14.2%) control cycles and 32/310 (10.3%) PRP cycles were contributed by women with more than one included cycle (**Supplementary Table 6**). Baseline demographic and reproductive characteristics were generally comparable between groups. Mean age was 27.7 ± 6.5 years in the control group and 27.5 ± 6.4 years in the PRP group, while mean BMI was 21.6 ± 5.0 and 22.1 ± 5.2 kg/m², respectively. Baseline endometrial thickness, pre-procedure endometrial thickness, ovarian reserve markers, thyroid-stimulating hormone, prolactin, and embryo-transfer characteristics were also similar between groups. Embryo-related transfer characteristics were also broadly comparable between groups, including embryo stage at transfer [cleavage-stage transfer: 256/310 (82.6%) in controls vs 271/310 (87.4%) in PRP; P = 0.115], euploid embryo transfer status [273/310 (88.1%) vs 279/310 (90.0%); P = 0.520], and overall PGT type distribution (P = 0.079) (**Table 1**).

**Table 1:** Baseline and cycle characteristics by study group; baseline demographic, reproductive, uterine, laboratory, and embryo-transfer characteristics stratified by intrauterine PRP exposure.

Characteristic	Control, n=310	PRP, n=310	P value	SMD
Age, years	27.7 ± 6.5	27.5 ± 6.4	0.728	0.03
BMI, kg/m²	21.6 ± 5.0	22.1 ± 5.2	0.274	0.09
Gravidity, n	1 (1–2)	2 (1–2)	0.115	0.13
Parity, n	1 (0–1)	1 (0–2)	0.049	0.14
Prior live births, n	1 (0–1)	1 (0–2)	0.033	0.16
Infertility duration, years	4 (1–8)	4 (1–7)	0.382	0.08
Prior IVF/ICSI cycles, n	4 (0–7)	6 (3–8)	<0.001	0.34
Prior ET cycles, n	1 (0–2)	1 (0–2)	0.823	0.00
Prior failed implantations, n	1 (0–2)	1 (0–2)	0.083	0.14
Prior miscarriages, n	1 (0–2)	1 (0–2)	0.483	0.06
Prior uterine surgery	102 (32.9)	114 (36.8)	0.312	0.08
AMH, ng/mL	6.8 ± 2.0	6.6 ± 2.0	0.263	0.09
Day-3 FSH, IU/L	4.9 ± 1.1	4.8 ± 1.2	0.289	0.09
TSH, mIU/L	2.48 ± 1.06	2.37 ± 1.00	0.194	0.10
Prolactin, ng/mL	12.6 ± 4.1	13.0 ± 4.5	0.260	0.09
Baseline endometrial thickness, mm	6.55 ± 2.53	6.53 ± 2.50	0.953	0.00
Pre-procedure endometrial thickness, mm	7.04 ± 2.91	6.89 ± 2.69	0.489	0.06
Uterine artery PI	3.12 ± 1.38	3.26 ± 1.29	0.187	0.11
Subendometrial flow VFI index	0.48 ± 0.29	0.50 ± 0.30	0.459	0.06
Thin endometrium	178 (57.4)	184 (59.4)	0.625	0.04
Clinician-recorded recurrent implantation failure^a^	146 (47.1)	181 (58.4)	0.005	0.23
History of adenomyosis	53 (17.1)	36 (11.6)	0.052	0.16
History of cavity-distorting fibroid	65 (21.0)	115 (37.1)	<0.001	0.36
History of hydrosalpinx	23 (7.4)	40 (12.9)	0.024	0.18
History of chronic endometritis	15 (4.8)	20 (6.5)	0.384	0.07
Frozen ET cycle	247 (79.7)	249 (80.3)	0.841	0.02
ICSI fertilization	225 (72.6)	211 (68.1)	0.218	0.10
Elective SET	112 (36.1)	114 (36.8)	0.867	0.01
Embryo stage at transfer (cleavage stage)	256 (82.6)	271 (87.4)	0.115	0.136
At least one embryo with euploid status recorded as transferred	273 (88.1)	279 (90.0)	0.520	0.062
PGT type (overall distribution)			0.079	0.203
PGT-A	109 (35.2)	127 (41.0)		0.120
PGT-M	77 (24.8)	86 (27.7)		0.066
PGT-SR	82 (26.5)	56 (18.1)		0.203
Unknown	42 (13.5)	41 (13.2)		0.009

^a^ RIF status was extracted from the medical record. Prior ET-cycle variables reflect the transfer-history data available in the center database and not necessarily complete lifetime transfer history.

Values are mean ± SD, median (IQR), or n (%). SMD = standardized mean difference. P values are from Welch t test, Mann–Whitney U test, chi-square test, or Fisher exact test as appropriate. AMH, anti-Müllerian hormone; BMI, body mass index; ET, embryo transfer; FSH, follicle-stimulating hormone; ICSI, intracytoplasmic sperm injection; IQR, interquartile range; IVF, *in vitro* fertilization; PI, pulsatility index; PRP, platelet-rich plasma; SD, standard deviation; SET, single-embryo transfer; SMD, standardized mean difference; TSH, thyroid-stimulating hormone; VFI, vascularization flow index.

However, several clinically relevant baseline differences were observed. The PRP group had a higher median number of prior IVF/ICSI cycles than the control group [6 (IQR, 3–8) vs. 4 (IQR, 0–7), P<0.001]. Clinician-recorded recurrent implantation failure was also more frequent in the PRP group than in the control group [181/310 (58.4%) vs. 146/310 (47.1%), P = 0.005]. In addition, history of cavity-distorting fibroid [115/310 (37.1%) vs. 65/310 (21.0%), P<0.001] and history of hydrosalpinx [40/310 (12.9%) vs. 23/310 (7.4%), P = 0.024] were more common among PRP-treated cycles. These variables were later considered as relevant covariates in the adjusted analyses (**Table 1**).

### PRP procedural characteristics

Among the 310 PRP-treated cycles, leukocyte-rich PRP was used in 246 cycles (79.4%), while pure PRP was used in 64 cycles (20.6%). The most common recorded PRP indication was combined thin endometrium and recurrent implantation failure, present in 111 cycles (35.8%), followed by thin endometrium alone in 73 cycles (23.5%) and recurrent implantation failure alone in 70 cycles (22.6%). PRP indication was unknown in 56 cycles (18.1%). The median PRP volume per infusion was 0.7 mL (IQR, 0.4–1.0), the median number of PRP infusions was 1 (IQR, 1–2), and the median interval from the last PRP infusion to embryo transfer was 2 days (IQR, 1–3). The median absolute PRP platelet concentration was 1301.5 (IQR, 924.25–1798.5) platelets/µL, corresponding to a median 5.04-fold enrichment over baseline peripheral platelet count (IQR, 3.89–6.33). PRP-related adverse events were recorded in 9 cycles (2.9%), including transient lower abdominal cramping in four, procedure-related pain in three cycles and later-onset infectious symptoms in two cycles despite antibiotic therapy. All of them were mild and did not lead to a delay in treatment (**Table 2**).

**Table 2:** Procedural characteristics of intrauterine PRP exposure among PRP-treated cycles.

PRP characteristic		PRP group, n=310
PRP type	Leukocyte-rich PRP	246 (79.4)
	Pure PRP	64 (20.6)
Primary PRP indication	Thin endometrium	73 (23.5)
	RIF	70 (22.6)
	Thin endometrium + RIF	111 (35.8)
	Unknown	56 (18.1)
PRP volume per infusion, mL		0.7 (0.4–1.0)
Number of PRP infusions		1 (1–2)
Days from last PRP to ET		2 (1–3)
PRP platelet concentration, absolute (platelets/μL)		1301.5 (924.25–1798.5)
PRP platelet concentration, × baseline		5.04 (3.89–6.33)
PRP adverse events		9 (2.9)

Values are median (IQR) or n (%). ET, embryo transfer; IQR, interquartile range; PRP, platelet-rich plasma; RIF, recurrent implantation failure; mL, milliliter.

### Endometrial response

Baseline endometrial thickness was similar between the control and PRP groups (6.55 ± 2.53 mm vs. 6.53 ± 2.50 mm; P = 0.953), as was pre-procedure/corresponding interim monitoring stage (7.04 ± 2.91 mm vs. 6.89 ± 2.69 mm; P = 0.489). At the post-procedure/corresponding subsequent monitoring stage, endometrial thickness was significantly greater in the PRP group than in the control group (9.18 ± 2.88 mm vs. 8.45 ± 3.40 mm; mean difference, 0.73 mm; 95% CI, 0.24 to 1.23; P = 0.004). The increase from the pre- to post-procedure monitoring stage was also greater in the PRP group (2.30 ± 3.45 mm vs. 1.41 ± 4.55 mm; mean difference, 0.89 mm; 95% CI, 0.25 to 1.53; P = 0.006) (**Table 3**).

**Table 3:** Endometrial response by PRP exposure; endometrial thickness and endometrial pattern according to PRP exposure.

Parameter	Control, n=310	PRP, n=310	PRP–control difference (95% CI)	P value
Baseline endometrial thickness, mm	6.55 ± 2.53	6.53 ± 2.50	−0.01 (−0.41 to 0.38)	0.953
Pre-procedure endometrial thickness, mm	7.04 ± 2.91	6.89 ± 2.69	−0.16 (−0.60 to 0.29)	0.489
Post-procedure endometrial thickness, mm	8.45 ± 3.40	9.18 ± 2.88	0.73 (0.24 to 1.23)	0.004
Change from pre- to post-procedure timepoint, mm	1.41 ± 4.55	2.30 ± 3.45	0.89 (0.25 to 1.53)	0.006
Endometrial thickness on progesterone start, mm	8.44 ± 3.04	9.09 ± 2.39	0.65 (0.22 to 1.08)	0.003
Endometrial thickness on ET day, mm	9.48 ± 2.73	10.58 ± 2.61	1.11 (0.68 to 1.53)	<0.001
Triple-line pattern near ET	136 (43.9)	211 (68.1)	24.2% (16.6 to 31.8)	<0.001

In control cycles, corresponding timepoints refer to analogous routine ultrasound monitoring stages within the standard cycle-preparation pathway. Values are mean ± SD or n (%). Between-group differences are PRP minus control. Endometrial thicknesses were analyzed using Welch t test and Endometrial pattern was analyzed using Pearson chi-square test. ANCOVA, analysis of covariance; CI, confidence interval; ET, embryo transfer; PRP, platelet-rich plasma; SD, standard deviation; mm, millimeter.

As a result, endometrial thickness on the day of progesterone start was significantly greater in the PRP group (9.09 ± 2.39 mm vs. 8.44 ± 3.04 mm; mean difference, 0.65 mm, 95% CI, 0.22 to 1.08, P = 0.003). Similarly, on embryo-transfer day, the PRP group had significantly greater endometrial thickness than the control group (10.58 ± 2.61 mm vs. 9.48 ± 2.73 mm; mean difference, 1.11 mm; 95% CI, 0.68 to 1.53; P<0.001). A triple-line endometrial pattern near embryo transfer was also more frequent in the PRP group [211/310 (68.1%) vs. 136/310 (43.9%); P<0.001] (**Table 3**).

In an ANCOVA model adjusted for pre-procedure endometrial thickness, PRP remained associated with a greater increase in endometrial thickness from the pre- to post-procedure monitoring stage, with an adjusted mean difference of 0.75 mm (95% CI, 0.25 to 1.24; P = 0.003). In repeated-measures mixed ANOVA across baseline, pre-procedure, post-procedure, progesterone-start, and embryo-transfer-day timepoints, there was a significant effect of monitoring stage (P<0.001) and a significant group × monitoring stage interaction (P<0.001), indicating that the pattern of endometrial thickness across routine monitoring stages differed between PRP-treated and control cycles. Greenhouse–Geisser correction did not materially change the conclusion.

### Unadjusted pregnancy outcomes

In unadjusted analyses, pregnancy outcomes were generally more favorable in the PRP group than in the control group. Biochemical pregnancy occurred in 105/310 PRP cycles (33.9%) and 87/310 control cycles (28.1%), but this difference did not reach statistical significance (RR, 1.21; 95% CI, 0.95–1.53; P = 0.118). Clinical pregnancy occurred in 98/310 PRP cycles (31.6%) and 64/310 control cycles (20.6%) (RR, 1.53; 95% CI, 1.17–2.01; P = 0.002). Ongoing pregnancy at 12 weeks occurred in 87/310 PRP cycles (28.1%) and 60/310 control cycles (19.4%) (RR, 1.45; 95% CI, 1.09–1.94; P = 0.011). Live birth occurred in 82/310 PRP cycles (26.5%) compared with 53/310 control cycles (17.1%), corresponding to an RR of 1.55 (95% CI, 1.14–2.10; P = 0.005). Ectopic pregnancy remained uncommon in both groups and was not significantly different. (**Table 4**).

**Table 4:** Unadjusted pregnancy outcomes by PRP exposure after embryo transfer.

Outcome	Control, n/N (%)	PRP, n/N (%)	RR (95% CI)	P value
Biochemical pregnancy	87/310 (28.1)	105/310 (33.9)	1.21 (0.95 to 1.53)	0.118
Clinical pregnancy	64/310 (20.6)	98/310 (31.6)	1.53 (1.17 to 2.01)	0.002
Ongoing pregnancy at 12 weeks	60/310 (19.4)	87/310 (28.1)	1.45 (1.09 to 1.94)	0.011
Live birth	53/310 (17.1)	82/310 (26.5)	1.55 (1.14 to 2.10)	0.005
Ectopic pregnancy	14/310 (4.5)	7/310 (2.3)	0.50 (0.20 to 1.22)	0.120
Multiple pregnancy	8/310 (2.6)	7/310 (2.3)	0.88 (0.32 to 2.38)	0.794

Risk ratios compare PRP with control. All rows were analyzed using Pearson chi-square test, except Multiple pregnancy (Fisher exact test was used). n/N, number of events/number of cycles. CI, confidence interval; ET, embryo transfer; PRP, platelet-rich plasma; RR, risk ratio.

To provide clinically contextualized denominators, ectopic pregnancy was also examined among cycles with biochemical pregnancy. Ectopic pregnancy occurred in 14/87 control group pregnancies (16.1%) and 7/105 PRP group pregnancies (6.7%). Multiple pregnancy was also examined among established pregnancies. Among clinical pregnancies, multiple pregnancy occurred in 8/64 control cycles (12.5%) and 7/98 PRP cycles (7.1%); among ongoing pregnancies at 12 weeks, the corresponding proportions were 8/60 (13.3%) and 7/87 (8.0%), respectively (**Supplementary Table 14**).

### Adjusted pregnancy-outcome models

After adjustment for age, BMI, prior IVF/ICSI cycles, prior ET cycles, thin endometrium, recurrent implantation failure, history of cavity-distorting fibroid, history of hydrosalpinx, pre-procedure endometrial thickness, number of embryos transferred, frozen embryo-transfer status, and PGT type, PRP remained associated with improved pregnancy outcomes. The adjusted RR for biochemical pregnancy was 1.18 (95% CI, 0.91–1.52; P = 0.205), indicating that the association for biochemical pregnancy was no longer statistically significant after adjustment. In contrast, PRP remained significantly associated with clinical pregnancy (adjusted RR, 1.51; 95% CI, 1.13–2.03; P = 0.005), ongoing pregnancy at 12 weeks (adjusted RR, 1.43; 95% CI, 1.05–1.95; P = 0.022), and live birth (adjusted RR, 1.55; 95% CI, 1.12–2.15; P = 0.009). Logistic-regression models yielded directionally consistent results, with an adjusted OR of 1.78 (95% CI, 1.18–2.70; P = 0.006) for live birth. (**Table 5**).

**Table 5:** Adjusted association between PRP and pregnancy outcomes; multivariable models estimating the association between intrauterine PRP infusion and pregnancy outcomes.

Outcome	Adjusted RR (95% CI)	P value	Adjusted OR (95% CI)	P value
Biochemical pregnancy	1.18 (0.91 to 1.52)	0.205	1.28 (0.89 to 1.84)	0.188
Clinical pregnancy	1.51 (1.13 to 2.03)	0.005	1.78 (1.21 to 2.63)	0.004
Ongoing pregnancy at 12 weeks	1.43 (1.05 to 1.95)	0.022	1.62 (1.09 to 2.42)	0.018
Live birth	1.55 (1.12 to 2.15)	0.009	1.78 (1.18 to 2.70)	0.006

Adjusted RR was estimated using modified Poisson regression with robust standard errors. Adjusted OR was estimated using logistic regression. Models adjusted for age, BMI, prior IVF/ICSI cycles, prior ET cycles, thin endometrium, RIF, history of cavity-distorting fibroid, history of hydrosalpinx, pre-procedure endometrial thickness, number of embryos transferred, frozen ET, and PGT type. BMI, body mass index; CI, confidence interval; ET, embryo transfer; ICSI, intracytoplasmic sperm injection; IVF, *in vitro* fertilization; OR, odds ratio; PGT, preimplantation genetic testing; PRP, platelet-rich plasma; RIF, recurrent implantation failure; RR, risk ratio; SE, standard error.

### Subgroup analyses for live birth

Exploratory subgroup analyses showed that the association between PRP and live birth was directionally consistent across most clinically relevant subgroups. Among cycles with thin endometrium, live birth occurred in 44/184 PRP cycles (23.9%) and 29/178 control cycles (16.3%), corresponding to an RR of 1.47 (95% CI, 0.96 to 2.24; P = 0.071). Among cycles without thin endometrium, the corresponding RR was 1.66 (95% CI, 1.06 to 2.60; P = 0.024), with no evidence of interaction by thin-endometrium status (interaction P = 0.697) (**Table 6**).

**Table 6:** Subgroup analyses for live birth by intrauterine PRP exposure with formal interaction tests for potential effect modifiers of the association between PRP and live birth.

Subgroup		Control	PRP	Unadjusted RR (95% CI)	P value	Interaction P value
Thin endometrium	Yes	29/178 (16.3)	44/184 (23.9)	1.47 (0.96 to 2.24)	0.071	0.697
	No	24/132 (18.2)	38/126 (30.2)	1.66 (1.06 to 2.60)	0.024	
RIF	Yes	24/146 (16.4)	50/181 (27.6)	1.68 (1.09 to 2.60)	0.016	0.570
	No	29/164 (17.7)	32/129 (24.8)	1.40 (0.90 to 2.19)	0.136	
Pre-procedure endometrium	<7 mm	29/160 (18.1)	52/180 (28.9)	1.59 (1.07 to 2.38)	0.020	0.755
	≥7 mm	24/150 (16.0)	30/130 (23.1)	1.44 (0.89 to 2.34)	0.134	
Number of transferred embryos	Single	28/198 (14.1)	38/183 (20.8)	1.47 (0.94 to 2.29)	0.088	0.859
	≥2	25/112 (22.3)	44/127 (34.6)	1.55 (1.02 to 2.36)	0.036	
Type of ET	Frozen	46/247 (18.6)	62/249 (24.9)	1.34 (0.95 to 1.88)	0.090	0.070
	Fresh	7/63 (11.1)	20/61 (32.8)	2.95 (1.35 to 6.47)	0.003	
Age	<35 years	40/253 (15.8)	68/257 (26.5)	1.67 (1.18 to 2.37)	0.003	0.332
	≥35 years	13/57 (22.8)	14/53 (26.4)	1.16 (0.60 to 2.23)	0.660	

Risk ratios compare PRP with control within each subgroup. P values are from Pearson chi-square test within-subgroups. Interaction P values were estimated using modified Poisson regression with robust standard errors. CI, confidence interval; ET, embryo transfer; PRP, platelet-rich plasma; RIF, recurrent implantation failure; RR, risk ratio; mm, millimeter.

Similarly, the association between PRP and live birth was observed both among cycles with recurrent implantation failure (RR, 1.68; 95% CI, 1.09 to 2.60; P = 0.016) and among cycles without recurrent implantation failure (RR, 1.40; 95% CI, 0.90 to 2.19; P = 0.136), with no significant interaction (interaction P = 0.570). When stratified by pre-procedure endometrial thickness, the association appeared stronger among cycles with endometrial thickness <7 mm (RR, 1.59; 95% CI, 1.07 to 2.38; P = 0.02) than among those with endometrial thickness ≥7 mm (RR, 1.44; 95% CI, 0.89 to 2.34; P = 0.134), although the interaction did not reach statistical significance (interaction P = 0.755) (**Table 6**).

The association between PRP and live birth was also present in both single-embryo and multiple-embryo transfer strata, with no significant interaction by number of embryos transferred (interaction P = 0.859). In analyses stratified by embryo-transfer type, the association between PRP and live birth was directionally positive in both frozen and fresh ET cycles. In frozen ET cycles, live birth occurred in 62/249 PRP cycles (24.9%) and 46/247 control cycles (18.6%), corresponding to an unadjusted RR of 1.34 (95% CI, 0.95–1.88; P = 0.090). In fresh ET cycles, live birth occurred in 20/61 PRP cycles (32.8%) and 7/63 control cycles (11.1%), corresponding to an unadjusted RR of 2.95 (95% CI, 1.35–6.47; P = 0.003). However, after multivariable adjustment, the fresh ET estimate was attenuated and no longer statistically significant (adjusted RR, 1.92; 95% CI, 0.84–4.40; P = 0.122), and the interaction between PRP exposure and ET type was no longer statistically significant (P for interaction=0.070) (**Supplementary Table 9**). In addition, fresh PRP and fresh control cycles differed in important transfer-related characteristics, including number of embryos transferred, and single-embryo transfer frequency. (**Supplementary Table 8**), however, the estimate remained similar after multivariable adjustments (**Supplementary Table 9**). The association was directionally similar in women aged <35 and ≥35 years, with no significant interaction by age group (interaction P = 0.332) (**Table 6**).

### Sensitivity analyses

Sensitivity analyses supported the robustness of the main live-birth finding. The primary adjusted modified Poisson model estimated an adjusted RR of 1.55 (95% CI, 1.12 to 2.15; P = 0.009), while the corresponding logistic-regression model estimated an adjusted OR of 1.78 (95% CI, 1.18–2.70; P = 0.006). Excluding PRP cycles with unknown indication yielded a similar estimate (RR, 1.56; 95% CI, 1.09–2.24; P = 0.016). The association also remained directionally consistent after excluding cycles with history of hydrosalpinx (RR, 1.54; 95% CI, 1.10–2.17; P = 0.012) and excluding cycles with history of cavity-distorting fibroid (RR, 1.83; 95% CI, 1.26–2.66; P = 0.001). In restricted subgroup analyses, the estimate remained directionally positive but was no longer statistically significant in frozen ET cycles (RR, 1.39; 95% CI, 0.97–2.01; P = 0.075), fresh ET cycles (RR, 1.92; 95% CI, 0.84–4.40; P = 0.122), and PGT-A cycles (RR, 1.47; 95% CI, 0.86–2.49; P = 0.156). The strongest restricted analysis remained among cycles with pre-procedure endometrial thickness <7 mm (RR, 1.57; 95% CI, 1.01–2.43; P = 0.043), in contrast, among cycles with pre-procedure endometrial thickness ≥7 mm, the association was directionally positive but not statistically significant (RR, 1.4; 95% CI, 0.86 to 2.29; P = 0.172) (**Supplementary Table 1**).

To address possible within-patient clustering, we repeated the adjusted live-birth analysis using patient-level cluster-robust standard errors and a GEE Poisson model. The estimated association between PRP and live birth remained materially unchanged in both analyses (cluster-robust modified Poisson: adjusted RR, 1.55, 95% CI, 1.12–2.15, P = 0.009); (GEE Poisson: adjusted RR, 1.55, 95% CI, 1.12–2.14, P = 0.008). Similarly, in the restriction analysis limited to women contributing only one ET cycle, PRP remained associated with live birth [adjusted RR, 1.61 (95% CI, 1.14–2.27, P = 0.007) (**Supplementary Table 7**).

In the IPTW sensitivity analysis, the estimated association between PRP and live birth remained similar to the primary adjusted model (weighted RR, 1.56; 95% CI, 1.16–2.11; P = 0.004) (**Supplementary Table 1**). The propensity-score model included age, BMI, prior IVF/ICSI cycles, prior ET cycles, thin endometrium, RIF status, cavity-distorting fibroid, hydrosalpinx, pre-procedure endometrial thickness, number of embryos transferred, embryo-transfer type, and PGT type. Covariate balance improved substantially after weighting, with all post-weighting standardized mean differences <0.10 (maximum 0.024) (**Supplementary Table 10**). Stabilized weights were truncated at the 1st and 99th percentiles in 14/620 cycles (2.3%), and overlap diagnostics suggested acceptable common support (**Supplementary Tables 11–13**).

### PRP procedural factors and live birth among PRP-treated cycles

Among PRP-treated cycles, procedural characteristics were not clearly associated with live-birth outcome. Pure PRP was used in 19/82 cycles (23.2%) with live birth and 45/228 cycles (19.7%) without live birth (P = 0.510). Median PRP volume, number of PRP infusions, interval from last PRP infusion to embryo transfer, and PRP platelet concentration were similar between cycles with and without live birth. PRP adverse events were uncommon in both groups. The distribution of PRP indication also did not differ significantly according to live-birth outcome (**Supplementary Table 2**).

### Conditional pregnancy progression and delivery outcomes

Among pregnancies classified as ongoing at 12 weeks, 53/60 (88.3%) in the control group and 82/87 (94.3%) in the PRP group resulted in live birth. In conditional analyses, progression from biochemical to clinical pregnancy was more frequent in the PRP group [98/105 (93.3%)] than in the control group [64/87 (73.6%)] (RR, 1.27; 95% CI, 1.11–1.45; P<0.001). Progression from clinical pregnancy to ongoing pregnancy at 12 weeks was similar between groups [87/98 (88.8%) vs. 60/64 (93.8%); RR, 0.95; 95% CI, 0.86–1.04; P = 0.408], as was progression from ongoing pregnancy at 12 weeks to live birth [82/87 (94.3%) vs. 53/60 (88.3%); RR, 1.07; 95% CI, 0.96–1.19; P = 0.230]. (**Supplementary Table 3**).

Among live-birth pregnancies, delivery and neonatal outcomes were analyzed descriptively and additionally stratified by singleton versus multiple live births. Overall, gestational age at delivery did not differ significantly between groups (39.5 ± 2.4 vs. 39.8 ± 2.0 weeks, P = 0.516). Mean birthweight was higher in the PRP group than in controls (3795.0 ± 222.6 vs. 3663.8 ± 237.8 g, P = 0.015); this difference remained present in the singleton-only analysis (3791.0 ± 214.3 vs. 3667.1 ± 230.6 g, P = 0.027). Preterm birth <37 weeks occurred in 5/29 (17.2%) control and 8/65 (12.3%) PRP pregnancies with available gestational-age data, while very preterm birth <34 weeks was not observed in either group. Low birthweight <2500 g was not observed in pregnancies with available birthweight records in either group. Because these analyses were restricted to live births, based on incomplete obstetric/neonatal follow-up for some pregnancies, and included few multiple births, they were interpreted as descriptive rather than as definitive safety comparisons (**Supplementary Table 4**).

## Discussion

In this cohort of 620 embryo-transfer cycles, intrauterine platelet-rich plasma (PRP) infusion before embryo transfer was associated with both improved endometrial development and higher pregnancy success. Although the PRP group had a less favorable baseline reproductive profile, including more prior IVF/ICSI cycles, recurrent implantation failure (RIF), history of cavity-distorting fibroid, and hydrosalpinx, PRP exposure remained independently associated with clinical pregnancy, ongoing pregnancy, and live birth after multivariable adjustment. The most clinically relevant finding was the persistent association between PRP exposure and live birth per embryo-transfer cycle.

The endometrial findings provide a biologically coherent link to the clinical outcomes. Baseline and pre-procedure endometrial thickness were similar between groups, but post-procedure and embryo-transfer-day endometrial thickness were greater in PRP-treated cycles. The repeated-measures mixed ANOVA also showed a significant group × time interaction, indicating that endometrial-thickness trajectories differed between PRP and control cycles. These findings suggest that PRP may be associated not simply with a one-time difference in endometrial measurement, but with a more favorable pattern of endometrial development across the treatment cycle (11, 16, 17).

The subgroup and sensitivity analyses support a cautious but reasonably robust interpretation of the main finding. The association between PRP and live birth was directionally consistent across most clinically relevant subgroups, including thin and non-thin endometrium, RIF and non-RIF, single-embryo and multiple-embryo transfer, and age strata. The PGT-A–restricted analysis remained directionally positive but was attenuated and no longer statistically significant, which may reflect reduced sample size but also indicates that embryo-level confounding cannot be excluded. This is important because the binary variable indicating transfer of at least one good morphological-quality embryo was not highly discriminative in this cohort, and more granular embryo-level prognostic features were not uniformly available for all cycles. Accordingly, part of the observed association may relate to embryo competence or embryo selection factors not fully captured in the main model.

Our findings are broadly compatible with the recent PRP literature, which has generally suggested potential benefit but has also emphasized substantial uncertainty. Meta-analyses and prior clinical studies have reported possible improvements in endometrial thickness and reproductive outcomes, particularly in women with thin endometrium or previous implantation failure (12–14, 18). At the same time, recent critical appraisals have emphasized that the available evidence remains limited by low-quality primary studies, heterogeneous RIF definitions, and variable PRP protocols (3, 8, 18, 19). Our data add real-world observational evidence using live birth as the primary outcome and incorporating multiple adjusted and sensitivity analyses, but they should still be interpreted as showing association rather than proof of efficacy.

Our work adds to the literature in several ways. First, it evaluates live birth as the main clinically meaningful outcome rather than relying only on endometrial thickness, biochemical pregnancy, or clinical pregnancy (1, 4). Second, it analyzes a relatively large and balanced cohort of PRP and control cycles. Third, it adjusts for important baseline and cycle-level confounders, including prior IVF/ICSI cycles, RIF, thin endometrium, history of hydrosalpinx, history of cavity-distorting fibroid, pre-procedure endometrial thickness, number of embryos transferred, frozen ET, and PGT type. Fourth, it links endometrial response, pregnancy outcomes, subgroup analyses, and sensitivity analyses giving a more reliable clinical picture.

The observed endometrial response is biologically plausible. PRP contains platelet-derived growth factors and cytokines that may influence tissue repair, angiogenesis, stromal remodeling, and inflammatory signaling, and prior mechanistic studies have suggested effects on endometrial stromal-cell proliferation, migration, regeneration-related pathways, and local immune response (11, 16, 17). While the present study is not mechanism-focused, the temporal pattern of the data is consistent with an association between PRP exposure and more favorable endometrial development across the treatment cycle.

From a clinical perspective, these findings suggest that PRP may be more relevant as a selective adjunct than as a universal implantation enhancer. The strongest signal in this cohort was observed among cycles with pre-procedure endometrial thickness <7 mm, which is the setting in which an endometrial-targeted intervention is most biologically intuitive. Even so, the present study should not be used to justify indiscriminate PRP use in all embryo-transfer cycles. Decisions regarding PRP use remain clinically nuanced and must be interpreted alongside embryo-related factors, euploidy status, uterine pathology, hydrosalpinx management, chronic endometritis, transfer technique, luteal support, and the patient’s cumulative reproductive history (3, 8, 19). The current findings therefore support further careful evaluation and selective clinical consideration rather than routine use.

Conditional pregnancy analyses suggested that the association of PRP with pregnancy success was driven mainly by higher progression from biochemical to clinical pregnancy, whereas progression from clinical pregnancy to ongoing pregnancy and from ongoing pregnancy to live birth was similar between groups. This pattern suggests that the observed association may be concentrated earlier in pregnancy establishment rather than clearly extending to later conditional progression after clinical pregnancy (2, 11, 16, 17).

Adverse events recorded after PRP were uncommon. Delivery and neonatal outcomes among live births were descriptive and did not show a clear difference in gestational age at delivery between groups. Birthweight was higher among live births in the PRP group, but this result should be interpreted cautiously because it was evaluated only among cycles resulting in live birth. Such conditional analyses are vulnerable to selection and collider bias and should not be interpreted as evidence that PRP improves neonatal growth (20). The recent meta-analysis in RIF also noted uncertainty around obstetric outcomes and raised the possibility that preterm birth findings require further investigation (18, 19). Larger studies with complete obstetric and neonatal follow-up are needed before any conclusion can be drawn about perinatal safety.

This study has several strengths. It evaluated a relatively large and balanced cohort of PRP-exposed and non-PRP embryo-transfer cycles and used live birth as the primary clinically meaningful outcome. The analysis integrated intermediate biological measures with reproductive outcomes and framed the main outcomes according to standardized ART terminology, supporting comparability with other studies (1). Methodologically, the study incorporated multivariable adjustment, logistic and modified Poisson models, subgroup analyses, clustered sensitivity analyses, and IPTW, which together strengthen confidence that the main association was not explained solely by measured baseline imbalance. In addition, repeated endometrial monitoring across the treatment cycle provided a more informative description of endometrial response than a single cross-sectional measurement.

This study has several important limitations. First, it was a retrospective, non-randomized cohort study, and PRP was offered in a real-world clinical setting to women with poorer baseline reproductive prognosis. Although we used frequency matching, multivariable adjustment, IPTW, and clustered sensitivity analyses, residual confounding and confounding by indication cannot be excluded. Matching was performed at the group level rather than as individual pair matching, and treatment assignment may still have been influenced by clinician judgment, endometrial response, and perceived prognosis. In addition, some key variables were derived from routine clinical documentation rather than uniform study-level adjudication. This is particularly relevant for clinician-recorded RIF, which was not independently reconstructed from complete lifetime transfer history and embryo-quality records for all cycles and therefore may be heterogeneous in definition. A further limitation relates to the definition of corresponding endometrial monitoring stages. The pre- and post-procedure measurements in PRP cycles were compared with corresponding routine monitoring stages in control cycles rather than with exact calendar-day–matched assessments. Although both PRP-treated and control cycles followed the center’s routine ultrasound monitoring pathway, exact dates for all interim ultrasound visits were not uniformly retained in analyzable structured form, and interval-based comparisons from baseline to pre-procedure, pre- to post-procedure, post-procedure to progesterone start, and progesterone start to embryo transfer could therefore not be reconstructed reliably for all cycles. Accordingly, residual time-related bias related to monitoring interval, patient attendance, progesterone timing, or cycle-management decisions cannot be completely excluded. Several other potentially important confounders were also incompletely captured or unavailable in analyzable form, including more granular embryo-level prognostic factors such as detailed morphology or developmental kinetics, exact luteal-support regimen, transfer difficulty, uterine contractility, clinician-level practice patterns, infertility etiologies not fully structured in the database, lifestyle-related factors, and adherence to treatment and follow-up recommendations. Protocol-level heterogeneity may also have influenced the results: frozen ET cycles included natural and modified natural-cycle preparation, and fresh ET subgroup analyses could not be fully adjusted for stimulation-related variables. The fresh ET subgroup should therefore be interpreted as exploratory and not as stand-alone evidence of clinical effectiveness.

Second, limitations related to data completeness, exposure characterization, and safety should also be acknowledged. The analytical cohort was assembled after exclusion of cycles with missing essential exposure, outcome, or key covariate information. Although residual missingness was minimal within the final analyzed cohort, selection bias remains possible if excluded cycles differed systematically from included cycles. A formal included-versus-excluded comparison was not feasible because many excluded cycles lacked the same baseline and cycle-level variables required for meaningful comparison, and some exclusions occurred for reasons other than missingness. Structured telephone follow-up was used to complete outcome ascertainment when routine records were incomplete, which may have introduced recall or ascertainment error. Some women contributed more than one ET cycle; although repeated-cycle contribution was limited and sensitivity analyses using cluster-robust standard errors, GEE, and restriction to one cycle per woman yielded materially similar results, complete independence between cycles cannot be assumed. PRP protocols were also heterogeneous across cycles with respect to type, volume, number of infusions, platelet concentration, and timing before embryo transfer; indication was unknown in 56 PRP cycles, although exclusion of these cycles did not materially alter the main live-birth association. In addition, detailed product-characterization measures such as full leukocyte quantification, red-cell contamination, and batch-level quality-control data were not uniformly retained for all retrospective cases. Finally, the study did not capture all clinically relevant safety outcomes, and obstetric/neonatal analyses were descriptive only. Delivery and neonatal outcomes were restricted to live-birth pregnancies, some follow-up data were incomplete, birthweight in multiple pregnancies was recorded at the pregnancy level rather than uniformly at the infant level, and several important maternal and neonatal outcomes were not consistently available. These findings should therefore not be interpreted as definitive evidence regarding the perinatal safety of intrauterine PRP.

Despite these limitations, the findings may still have clinical relevance. Women considering PRP should be counseled that it remains an adjunctive intervention with promising but not definitive evidence; that available data suggest a possible association with improved endometrial development and better reproductive outcomes; and that any potential benefit may be greatest in selected women with suboptimal endometrial development rather than across all embryo-transfer cycles. This balanced counseling is particularly important because women with RIF or thin endometrium are often exposed to multiple add-on interventions with variable evidentiary support (8, 19).

## Conclusion

In this cohort study, intrauterine PRP infusion before embryo transfer was associated with improved endometrial-thickness trajectory and higher rates of clinical pregnancy, ongoing pregnancy, and live birth, while biochemical pregnancy showed a directionally positive but non-significant association. The association with live birth persisted after adjustment for baseline and cycle-level confounders and remained directionally consistent across sensitivity analyses. The strongest clinical signal was observed among cycles with pre-procedure endometrial thickness <7 mm, suggesting that PRP may be most relevant as a targeted adjunct for suboptimal endometrial development. Well-designed randomized trials with standardized PRP protocols, contemporary RIF definitions, live birth as the primary outcome, and complete obstetric/neonatal follow-up are needed before PRP can be recommended as routine practice.

## Data Availability

The original contributions presented in the study are included in the article/**Supplementary Material**, further inquiries can be directed to the corresponding author.
