# Supplementary material for: Association of intrauterine platelet-rich plasma infusion before embryo transfer with endometrial response and live birth: a cohort study
Source: Front Endocrinol (Lausanne). 2026 Aug 26;17:1863693. doi: 10.3389/fendo.2026.1863693 (PMC13553497; doi:10.3389/fendo.2026.1863693)
Supplement: Supplementary file 1 [file Table1.docx]

**Supplementary Table 1.** Sensitivity analyses for live birth evaluating the robustness of the association between intrauterine PRP and live birth.

| **Sensitivity analysis** | **N** | **Effect estimates** | **P value** |
| --- | --- | --- | --- |
| Unadjusted PRP vs control | 620 | 1.55 (1.14 to 2.10) | 0.005 |
| Primary adjusted model | 620 | 1.55 (1.12 to 2.15) | 0.009 |
| Same covariates, logistic model (OR) | 620 | 1.78 (1.18 to 2.70) | 0.006 |
| Excluding PRP cycles with unknown indication | 564 | 1.56 (1.09 to 2.24) | 0.016 |
| Excluding cycles with history of hydrosalpinx | 557 | 1.54 (1.10 to 2.17) | 0.012 |
| Excluding cycles with history of cavity-distorting fibroid | 440 | 1.83 (1.26 to 2.66) | 0.001 |
| Restricted to frozen ET cycles | 496 | 1.39 (0.97 to 2.01) | 0.075 |
| Restricted to fresh ET cycles | 124 | 1.92 (0.84 to 4.40) | 0.122 |
| Restricted to single-embryo transfer | 381 | 1.64 (1.03 to 2.60) | 0.037 |
| Restricted to ≥2 embryos transferred | 239 | 1.42 (0.93 to 2.16) | 0.100 |
| Restricted to pre-procedure endometrium <7 mm | 340 | 1.57 (1.01 to 2.43) | 0.043 |
| Restricted to pre-procedure endometrium ≥7 mm | 280 | 1.40 (0.86 to 2.29) | 0.172 |
| Restricted to known PGT-A cycles | 236 | 1.47 (0.86 to 2.49) | 0.156 |
| Stabilized IPTW, weights truncated 1st–99th percentile | 620 | 1.56 (1.16 to 2.11) | 0.004 |

The primary adjusted model included age, BMI, prior IVF/ICSI cycles, prior ET cycles, thin endometrium, RIF, history of cavity-distorting fibroid, history of hydrosalpinx, pre-procedure endometrial thickness, number of embryos transferred, fresh/frozen ET status, and PGT type. Modified Poisson robust Wald test or linear regression has been used, as appropriate. **Abbreviations**: BMI, body mass index; CI, confidence interval; ET, embryo transfer; ICSI, intracytoplasmic sperm injection; IPTW, inverse probability of treatment weighting; IVF, in vitro fertilization; OR, odds ratio; PGT-A, preimplantation genetic testing for aneuploidy; PRP, platelet-rich plasma; RIF, recurrent implantation failure; RR, risk ratio.

**Supplementary Table 2.** PRP procedural characteristics according to live birth among PRP-treated cycles

| **PRP-only variable** | **No live birth, n=228** | **Live birth, n=82** | **P value** |
| --- | --- | --- | --- |
| Pure PRP | 45/228 (19.7) | 19/82 (23.2) | 0.510 |
| PRP volume per infusion, mL | 0.70 (0.40–1.00) | 0.70 (0.40–1.00) | 0.577 |
| Number of PRP infusions | 1 (1–1) | 1 (1–1) | 0.378 |
| Days from last PRP to ET | 2 (1–3) | 2 (1–3) | 0.289 |
| PRP platelet concentration, × baseline | 5.07 (3.88–6.31) | 4.92 (4.02–6.52) | 0.946 |
| PRP adverse event | 8/228 (3.5) | 1/82 (1.2) | 0.454 |
| Indication: Thin endometrium | 56/228 (24.6) | 17/82 (20.7) | 0.483 |
| Indication: RIF | 47/228 (20.6) | 23/82 (28.0) | 0.167 |
| Indication: Thin endometrium + RIF | 84/228 (36.8) | 27/82 (32.9) | 0.526 |

Values are median (IQR) or n (%). P values are from Mann–Whitney U test and chi-square test as appropriate.

**Supplementary Table 3.** Conditional pregnancy progression after biochemical pregnancy among cycles reaching each preceding milestone.

| **Conditional outcome** | **Control** | **PRP** | **RR (95% CI)** | **P value** |
| --- | --- | --- | --- | --- |
| Biochemical to clinical pregnancy | 64/87 (73.6%) | 98/105 (93.3%) | 1.27 (1.11 to 1.45) | <0.001 |
| Clinical to ongoing pregnancy at 12 weeks | 60/64 (93.8%) | 87/98 (88.8%) | 0.95 (0.86 to 1.04) | 0.408 |
| Ongoing pregnancy at 12 weeks to live birth | 53/60 (88.3%) | 82/87 (94.3%) | 1.07 (0.96 to 1.19) | 0.230 |
| Biochemical pregnancy to live birth | 53/87 (60.9%) | 82/105 (78.1%) | 1.28 (1.05 to 1.56) | 0.010 |

Risk ratios compare PRP with control. **Abbreviations**: CI, confidence interval; PRP, platelet-rich plasma; RR, risk ratio.

**Supplementary Table 4.** Delivery and neonatal outcomes among cycles resulting in live birth

| **Section** | **Outcome** | **Control** | **PRP** | **P value** |
| --- | --- | --- | --- | --- |
| **All live births** | Live-birth pregnancies, n | 53 | 82 | — |
|  | Singleton live-birth pregnancies, n (%) | 45/53 (84.9) | 75/82 (91.5) | 0.236 |
|  | Multiple live-birth pregnancies, n (%) | 8/53 (15.1) | 7/82 (8.5) | 0.236 |
|  | Liveborn infants, total n | 61 | 89 | — |
|  | Gestational age at delivery, weeks, n/N | 39.5 ± 2.4 (n=29) | 39.8 ± 2.0 (n=65) | 0.516 |
|  | Birthweight, g, n/N | 3663.8 ± 237.8 (n=29) | 3795.0 ± 222.6 (n=65) | 0.015 |
|  | Preterm birth <37 weeks, n/N (%) | 5/29 (17.2) | 8/65 (12.3) | 0.531 |
|  | Very preterm birth <34 weeks, n/N (%) | 0/29 (0.0) | 0/65 (0.0) | 1.000 |
|  | Low birthweight <2500 g, n/N (%) | 0/29 (0.0) | 0/65 (0.0) | 1.000 |
|  | Macrosomia / high birthweight ≥4000 g, n/N (%) | 2/29 (6.9) | 11/65 (16.9) | 0.332 |
| **Singleton live births only** | Live-birth pregnancies, n | 45 | 75 | — |
|  | Gestational age at delivery, weeks, n/N | 39.5 ± 2.5 (n=25) | 39.8 ± 2.0 (n=58) | 0.523 |
|  | Birthweight, g, n/N | 3667.1 ± 230.6 (n=25) | 3791.0 ± 214.3 (n=58) | 0.027 |
|  | Preterm birth <37 weeks, n/N (%) | 5/25 (20.0) | 7/58 (12.1) | 0.497 |
|  | Very preterm birth <34 weeks, n/N (%) | 0/25 (0.0) | 0/58 (0.0) | 1.000 |
|  | Low birthweight <2500 g, n/N (%) | 0/25 (0.0) | 0/58 (0.0) | 1.000 |
|  | Macrosomia / high birthweight ≥4000 g, n/N (%) | 1/25 (4.0) | 9/58 (15.5) | 0.269 |
| **Multiple live births only** | Live-birth pregnancies, n | 8 | 7 | — |
|  | Gestational age at delivery, weeks, n/N | 39.5 ± 1.7 (n=4) | 39.6 ± 2.2 (n=7) | 0.954 |
|  | Birthweight, g, n/N | 3643.0 ± 319.0 (n=4) | 3828.1 ± 301.8 (n=7) | 0.381 |
|  | Preterm birth <37 weeks, n/N (%) | 0/4 (0.0) | 1/7 (14.3) | 1.000 |
|  | Very preterm birth <34 weeks, n/N (%) | 0/4 (0.0) | 0/7 (0.0) | 1.000 |
|  | Low birthweight <2500 g, n/N (%) | 0/4 (0.0) | 0/7 (0.0) | 1.000 |
|  | Macrosomia / high birthweight ≥4000 g, n/N (%) | 1/4 (25.0) | 2/7 (28.6) | 1.000 |

Delivery and neonatal outcomes were analyzed among live-birth pregnancies only. Gestational age and birthweight were not available for all live-birth pregnancies; therefore, denominators for preterm birth and birthweight-category outcomes are based on available records. In the dataset, birthweight for multiple live births was recorded as the average birthweight per pregnancy. Macrosomia/high birthweight was defined as ≥4000 g. P values are from Welch t test, Mann–Whitney U, Fisher exact test or Pearson chi-square test as appropriate. **Abbreviations**: CI, confidence interval; IQR, interquartile range; NA, not applicable; PRP, platelet-rich plasma; SD, standard deviation; g, gram.

**Supplementary Table 5. Frequency-matching strata and distribution of PRP and non-PRP cycles before and after control selection**

| **Matching stratum** | | **Eligible PRP cycles (n=310)** | **Eligible non-PRP cycles before selection (n=1529)** | **Selected control cycles after matching (n=310)** |
| --- | --- | --- | --- | --- |
| **Age categories** | <28 years | 175 (56.5%) | 669 (43.8%) | 189 (61%) |
|  | ≥28 years | 135 (43.5%) | 860 (56.2%) | 121 (39%) |
| **Baseline endometrial-thickness categories** | <7 mm | 185 (59.7%) | 743 (48.6%) | 181 (58.4%) |
|  | ≥7 mm | 125 (40.3%) | 786 (51.4%) | 129 (41.6%) |
| **Prior ET-cycle categories** | ≤1 cycle | 222 (71.6%) | 1254 (82%) | 220 (71%) |
|  | >1 cycles | 88 (28.4%) | 275 (18%) | 90 (29%) |

**Supplementary Table 6. Patient-level contribution of embryo-transfer cycles**

| **Patient-level characteristic** | **Value** | |
| --- | --- | --- |
| Total ET cycles | 620 | |
| Unique women | 582 | |
| Women contributing 1 cycle | 544 (93.5%) | |
| Women contributing 2 cycles | 38 (6.5%) | |
| Women contributing >2 cycles | 0 (0.0%) | |
| Women contributing both PRP and control cycles | 6 (1.0% of women; 15.8% of women with >1 cycle) | |
| **Cycle-level characteristic** | **Control** | **PRP** |
| Cycles contributed by women with >1 included cycle | 44/310 (14.2%) | 32/310 (10.3%) |
| Cycles contributed by women with only 1 included cycle | 266/310 (85.8%) | 278/310 (89.7%) |

**Supplementary Table 7. Sensitivity analyses accounting for within-patient clustering for association between PRP and live birth**

| **Model** | **N** | **Adjusted RR (95% CI) for PRP** | **P value** |
| --- | --- | --- | --- |
| Primary modified Poisson model | 620 cycles | 1.55 (1.12 to 2.15) | 0.009 |
| Modified Poisson with patient-level cluster-robust SE | 620 cycles | 1.55 (1.11 to 2.15) | 0.009 |
| GEE Poisson model with exchangeable correlation | 620 cycles | 1.55 (1.12 to 2.14) | 0.008 |
| Restricted to women contributing only 1 cycle | 582 women / cycles | 1.61 (1.14 to 2.27) | 0.007 |

**Supplementary Table 8. Baseline and cycle characteristics in fresh ET cycles by PRP exposure**

| **Characteristic** | **Control (n=63)** | **Fresh PRP (n=61)** | **P value** | **Control (n=247)** | **PRP (n=249)** | **P value** |
| --- | --- | --- | --- | --- | --- | --- |
|  | **Fresh ET** | | | **Frozen ET** | | |
| Age, years | 26.95 ± 6.38 | 27.38 ± 6.33 | 0.711 | 27.93 ± 6.49 | 27.59 ± 6.48 | 0.563 |
| BMI, kg/m² | 21.77 ± 4.97 | 23.53 ± 5.38 | 0.061 | 21.60 ± 5.03 | 21.72 ± 5.05 | 0.781 |
| Prior IVF/ICSI cycles, n | 4 (1–7) | 5 (2–8) | 0.066 | 4 (0–8) | 6 (3–9) | <0.001 |
| Prior ET cycles, n | 1 (0–2) | 1 (0–2) | 0.127 | 1 (0–2) | 1 (0–2) | 0.636 |
| Prior failed implantations, n | 1 (0–2) | 1 (0–2) | 0.600 | 1 (0–2) | 1 (0–2) | 0.028 |
| Baseline endometrial thickness, mm | 6.50 ± 2.34 | 6.33 ± 2.16 | 0.668 | 6.56 ± 2.58 | 6.59 ± 2.58 | 0.906 |
| Pre-procedure endometrial thickness, mm | 6.75 ± 2.90 | 6.76 ± 2.61 | 0.984 | 7.12 ± 2.91 | 6.92 ± 2.71 | 0.430 |
| Thin endometrium | 35/63 (55.6%) | 37/61 (60.7%) | 0.565 | 143/247 (57.9%) | 147/249 (59.0%) | 0.796 |
| Clinician-recorded RIF | 25/63 (39.7%) | 30/61 (49.2%) | 0.287 | 121/247 (49.0%) | 151/249 (60.6%) | 0.009 |
| History of cavity-distorting fibroid | 14/63 (22.2%) | 21/61 (34.4%) | 0.131 | 51/247 (20.6%) | 94/249 (37.8%) | <0.001 |
| History of hydrosalpinx | 5/63 (7.9%) | 7/61 (11.5%) | 0.505 | 18/247 (7.3%) | 33/249 (13.3%) | 0.029 |
| Embryos transferred, n | 1 (1–2) | 2 (1–3) | 0.003 | 1 (1–2) | 1 (1–2) | 0.774 |
| Single embryo transfer | 42/63 (66.7%) | 27/61 (44.3%) | 0.012 | 156/247 (63.2%) | 156/249 (62.7%) | 0.907 |
| ICSI | 49/63 (77.8%) | 44/61 (72.1%) | 0.468 | 176/247 (71.3%) | 167/249 (67.1%) | 0.313 |
| At least one embryo with euploid status recorded as transferred | 56/63 (88.9%) | 57/61 (93.4%) | 0.565 | 217/247 (87.9%) | 222/249 (89.2%) | 0.754 |
| Embryo stage at transfer (cleavage stage) | 54/63 (85.7%) | 51/61 (83.6%) | 0.939 | 202/247 (81.8%) | 220/249 (88.4%) | 0.054 |
| PGT type (overall distribution) | PGT-A: 21/63 (33.3%)  PGT-M: 18/63 (28.6%)  PGT-SR: 18/63 (28.6%)  Unknown: 6/63 (9.5%) | PGT-A: 25/61 (41.0%)  PGT-M: 21/61 (34.4%)  PGT-SR: 9/61 (14.8%)  Unknown: 6/61 (9.8%) | 0.315 | PGT-A: 88/247 (35.6%)  PGT-M: 59/247 (23.9%)  PGT-SR: 64/247 (25.9%) Unknown: 36/247 (14.6%) | PGT-A: 102/249 (41.0%)  PGT-M: 65/249 (26.1%)  PGT-SR: 47/249 (18.9%)  Unknown: 35/249 (14.1%) | 0.269 |

Embryo stage at transfer was classified as cleavage-stage for embryo day <5 and blastocyst for embryo day ≥5. At least one euploid embryo transferred was derived from embryo ploidy records (any transferred embryo coded as euploid).

**Supplementary Table 9. PRP effect on live birth in frozen and fresh ET subgroups**

| **Subgroup** | **Model** | **Detail** | **PRP effect on live birth (RR, 95% CI)** | **P value** |
| --- | --- | --- | --- | --- |
| Frozen ET | Unadjusted | 62/249 vs 46/247 | 1.34 (0.95 to 1.88) | 0.090 |
| Frozen ET | Adjusted primary | n=496 | 1.39 (0.97 to 2.01) | 0.075 |
| Frozen ET | Adjusted + embryo stage | n=496 | 1.37 (0.93 to 2.01) | 0.106 |
| Frozen ET | Adjusted + embryo stage + any euploid | n=496 | 1.37 (0.93 to 2.02) | 0.106 |
| Fresh ET | Unadjusted | 20/61 vs 7/63 | 2.95 (1.35 to 6.47) | 0.003 |
| Fresh ET | Adjusted primary | n=124 | 1.92 (0.84 to 4.40) | 0.122 |
| Fresh ET | Adjusted + embryo stage | n=124 | 1.77 (0.69 to 4.51) | 0.232 |
| Fresh ET | Adjusted + embryo stage + any euploid | n=124 | 1.75 (0.66 to 4.64) | 0.258 |

**Supplementary Table 10. Covariate balance before and after IPTW**

| **Covariate** | **Unweighted control** | **Unweighted PRP** | **Abs SMD before** | **Weighted control** | **Weighted PRP** | **Abs SMD after** |
| --- | --- | --- | --- | --- | --- | --- |
| Age, years | 27.73 | 27.55 | 0.028 | 27.75 | 27.82 | 0.009 |
| Body mass index, kg/m² | 21.63 | 22.08 | 0.088 | 21.81 | 21.82 | 0.002 |
| Prior IVF/ICSI cycles, n | 4.42 | 5.77 | 0.340 | 5.21 | 5.24 | 0.007 |
| Prior ET cycles, n | 0.98 | 0.98 | 0.004 | 0.95 | 0.95 | 0.005 |
| Thin endometrium | 57.4% | 59.4% | 0.039 | 58.0% | 58.5% | 0.010 |
| RIF status | 47.1% | 58.4% | 0.227 | 52.4% | 52.6% | 0.005 |
| History of Cavity-distorting fibroid | 21.0% | 37.1% | 0.360 | 28.7% | 29.7% | 0.024 |
| History of Hydrosalpinx | 7.4% | 12.9% | 0.182 | 10.0% | 10.2% | 0.010 |
| Pre-procedure endometrial thickness, mm | 7.04 | 6.89 | 0.056 | 7.04 | 6.97 | 0.024 |
| Embryos transferred, n | 1.50 | 1.61 | 0.152 | 1.57 | 1.56 | 0.012 |
| Fresh ET | 20.3% | 19.7% | 0.016 | 19.8% | 19.8% | 0.001 |
| PGT-A | 35.2% | 41.0% | 0.120 | 36.5% | 37.3% | 0.016 |
| PGT-M | 24.8% | 27.7% | 0.066 | 27.5% | 27.0% | 0.009 |
| PGT-SR | 26.5% | 18.1% | 0.202 | 22.5% | 22.5% | 0.000 |
| PGT unknown | 13.5% | 13.2% | 0.009 | 13.6% | 13.2% | 0.011 |

SMD = standardized mean difference.

**Supplementary Table 11. Distribution of stabilized IPTW weights** **before and after truncation at the 1st and 99th percentiles**

| **Weight set** | **Min** | **1st pct** | **5th pct** | **Median** | **95th pct** | **99th pct** | **Max** | **Mean ± SD** | **Overall ESS** |
| --- | --- | --- | --- | --- | --- | --- | --- | --- | --- |
| Raw stabilized weights | 0.565 | 0.602 | 0.643 | 0.889 | 1.664 | 2.342 | 4.842 | 1.005 ± 0.420 | 527.9 |
| Truncated stabilized weights | 0.602 | 0.602 | 0.643 | 0.889 | 1.664 | 2.342 | 2.342 | 0.992 ± 0.345 | 553.3 |

ESS: Effective sample size

**Supplementary Table 12. Truncation counts and effective sample size by group**

| **Group** | **N** | **Truncated below 1st pct** | **Truncated above 99th pct** | **Total truncated** | **% truncated** | **ESS after truncation** |
| --- | --- | --- | --- | --- | --- | --- |
| Overall | 620 | 7 | 7 | 14 | 2.3% | 553.3 |
| Control | 310 | 1 | 4 | 5 | 1.6% | 274.9 |
| PRP | 310 | 6 | 3 | 9 | 2.9% | 278.4 |

ESS: Effective sample size

**Supplementary Table 13. Propensity-score overlap by treatment group**

| **Group** | **Mean ± SD** | **Min** | **5th pct** | **Median** | **95th pct** | **Max** |
| --- | --- | --- | --- | --- | --- | --- |
| Control | 0.448 ± 0.151 | 0.161 | 0.247 | 0.431 | 0.706 | 0.897 |
| PRP | 0.552 ± 0.153 | 0.155 | 0.313 | 0.557 | 0.795 | 0.885 |

**Supplementary Table 14. Ectopic pregnancy and multiple pregnancy using clinically contextualized denominators**

| **Outcome** | **Control** | **PRP** | **P value** |
| --- | --- | --- | --- |
| Ectopic pregnancy per ET cycle | 14/310 (4.5%) | 7/310 (2.3%) | **0.182** |
| Ectopic pregnancy per biochemical pregnancy | 14/87 (16.1%) | 7/105 (6.7%) | **0.061** |
| Multiple pregnancy per ET cycle | 8/310 (2.6%) | 7/310 (2.3%) | **1.000** |
| Multiple pregnancy per clinical pregnancy | 8/64 (12.5%) | 7/98 (7.1%) | **0.277** |
| Multiple pregnancy per ongoing pregnancy at 12 weeks | 8/60 (13.3%) | 7/87 (8.0%) | **0.407** |

P values were calculated using two-sided Fisher’s exact test because of small event counts.
